# Advances in Behavioral Remote Data Collection in the Home Setting: Assessing the Mother-Infant Relationship and Infant’s Adaptive Behavior via Virtual Visits

**DOI:** 10.3389/fpsyg.2021.703822

**Published:** 2021-10-01

**Authors:** Eunkyung Shin, Cynthia L. Smith, Brittany R. Howell

**Affiliations:** ^1^Fralin Biomedical Research Institute at Virginia Tech Carilion, Roanoke, VA, United States; ^2^Department of Human Development and Family Science, Virginia Tech, Blacksburg, VA, United States

**Keywords:** remote data collection, behavioral observation, home setting, infant adaptive behavior, mother-infant relationship

## Abstract

Psychological science is struggling with moving forward in the midst of the COVID-19 pandemic, especially due to the halting of behavioral data collection in the laboratory. Safety barriers to assessing psychological behavior in person increased the need for remote data collection in natural settings. In response to these challenges, researchers, including our team, have utilized this time to advance remote behavioral methodology. In this article, we provide an overview of our group’s strategies for remote data collection methodology and examples from our research in collecting behavioral data in the context of psychological functioning. Then, we describe the design and development of our strategies for remote data collection of mother-infant interactions, with the goal being to assess maternal sensitivity and intrusiveness, as well as infants’ adaptive behaviors in several developmental domains. During these virtual visits over Zoom, mother-infant dyads watched a book-reading video and were asked to participate in peek-a-boo, toy play, and toy removal tasks. After the behavioral tasks, a semi-structured interview (Vineland Adaptive Behavior Scale – VABS III) was conducted to assess the infant’s adaptive behavior in communication, socialization, daily living skills, and motor domains. We delineate the specific strategies we applied to integrate laboratory tasks and a semi-structured interview into remote data collection in home settings with mothers and infants. We also elaborate on issues encountered during remote data collection and how we resolved these challenges. Lastly, to inform protocols for future remote data collection, we address considerations and recommendations, as well as benefits and future directions for behavioral researchers in developmental psychology research.

## Introduction

During the COVID-19 pandemic, investigators have faced challenges in conducting research, with traditional face-to-face data collection methods having been paused or otherwise disrupted. Social distancing mandates and safety barriers forced researchers to shift in person data collection in laboratories to remote data collection in other settings (Sy et al., 2020). Thus, observational measures were restricted during the COVID-19 pandemic. While this restriction caused many disruptions to traditional behavioral assessment data collection, this unique situation also forced researchers to consider novel research designs and to develop remote data collection methodology.

Despite challenges in data collection during this time of social distancing, advances in technology, such as the increased access to synchronous web-based video conferencing platforms (e.g., Zoom and Skype), have allowed for innovative ways of collecting behavioral data that may compensate for the lack of, or extend, traditional face-to-face data collection methods. Even prior to restrictions on in person data collection, remote data collection methods have been implemented in behavioral research (Strickland et al., 2003). However, most studies have predominantly focused on qualitative research including online interviews and focus groups (Archibald et al., 2019). Few reports have been published about observational data collection using web-based video conferencing platforms in naturalistic settings. The purpose of the current report is to share strategies and experiences in remote data collection in naturalistic settings using video conferencing platforms to enrich the methods available to collect behavioral data during this global health crisis. In this article, we delineate the specific strategies that we applied to integrate laboratory tasks and a semi-structured interview into remote data collection in home settings with mothers and infants. We also elaborate on issues encountered during remote data collection and how we resolved these challenges. Lastly, we address considerations and recommendations for behavioral researchers in child development research to inform optimized protocols for future remote data collection.

### Web-Based Data Collection

Remote data collection provides greater flexibility and effectiveness in time, cost, and access to participants. In qualitative research on participants’ perception about research using Skype (Lo Iacono et al., 2016), participants mentioned that they prefer taking part in research at home to traveling to the laboratory in terms of the amount of time that they spend for research. Participants can save time and the cost of traveling to the laboratory, and researchers can also have flexibility in timing and space where they conduct research. In particular, given that mother-infant interaction is more likely to be related to infant’s feeding and sleeping schedules, and/or child temperament characteristics that may make being in unfamiliar spaces stressful (Graag et al., 2012), comfortable space and thoughtful scheduling are necessary to accurately capture mother-infant interactions in daily life. Resolving these logistical issues allows researchers to access geographically diverse and disadvantaged populations, as long as researchers accommodate access to internet tools and environment (Sy et al., 2020). Thus, remote data collection can be used for rural populations and cross-cultural studies with better access to participants who face challenges to in-person participation, including reduced mobility or large geographical spread.

Two types of web-based data collection technology have been utilized in the past – asynchronous and synchronous (Berg, 2007). Asynchronous methods support web-based communication at different times such as email or online surveys. Scott and Schulz (2017) developed an asynchronous online platform called Lookit, to collect infants’ preferential looking paradigms. Parents participated in self-administered tasks with their children at their convenience by accessing the Lookit website without live interaction with researchers. Lookit is available for researchers to conduct their own research via Github Projects^1^. Recently, Rhodes et al. (2020) conducted unmoderated remote research in which parents and their children participated in online software using families’ webcams without involvement of researchers. Items about gender stereotypes and parent-child conversations about gender were conducted using the online software. A study setting without direct interaction with researchers putatively elicits more natural behavior from families because of the absence of strangers (Rhodes et al., 2020). Resources used for implementation of the study have been shared on the following website^2^.

In contrast, synchronous methods include real-time interactions such as online messengers and video conference calls (e.g., Zoom and Skype) that enable back-and-forth exchange of interactions (Sullivan, 2012). Sheskin and Keil (2018) developed a video chat platform to validate the method by replicating standard developmental tasks with children aged between 5 and 10 years old. Most children in their study presented correct answers in social tasks and causal reasoning tasks. Because synchronous methods transmit verbal and non-verbal cues through real time video and audio, researchers are better able to replicate the features of face-to-face in-person interactions using these technologies. We implemented a synchronous method because live interaction with researchers allows the researcher to conduct the study in a consistent way across all participants (Sheskin and Keil, 2018). Although few studies using online data collection have been published, there have been efforts to advance the field of online assessment. For example, “Many Babies-At Home^3^” is a methodological project in which researchers developed cross-cultural online testing of infants. In this project, multiple laboratories across the world have collaborated to develop and distribute universal and robust practices in online testing methods for developmental studies.

### Current Study

In this article, we describe the application of remote data collection in a natural home setting through a video conferencing platform to share our experiences with researchers who are considering new remote data collection methods. We provide an overview of our group’s strategies for remote data collection methodology and examples from our research in collecting behavioral data in the context of psychological functioning. Then, we describe the design and development of our strategies for remote data collection of mother-infant interactions, with the goal being to assess maternal sensitivity and intrusiveness, as well as infants’ adaptive behaviors in several developmental domains. We delineate the specific strategies we applied to integrate laboratory tasks and a semi-structured interview into remote data collection in home settings with mothers and infants. We also elaborate on issues encountered during remote data collection and how we resolved these challenges. Lastly, to inform protocols for future remote data collection, we address considerations and recommendations, as well as benefits and future directions for behavioral researchers in developmental psychology research.

## Implementation of Remote Data Collection

We originally planned to invite mothers and their infants to our lab to conduct a 10-min free play session in which mothers and infants interact with a standardized set of toys. Additionally, the Bayley Scales of Infant and Toddler Development – Fourth edition (Bayley-4; Bayley and Aylward, 2019) and episodes from the Laboratory Temperament Assessment Battery (Lab-TAB; Goldsmith et al., 1993) were planned to assess general development, temperament, and mother-infant behavioral interactions. After the outbreak of COVID-19, we had to shift our plan for face-to-face data collection in our laboratory to remote data collection. Given that synchronous web-based video conferencing platforms can capture real-time interaction with a private recording function, we decided to collect behavioral data through a video conferencing platform, specifically Zoom^4^. The remote data collection for our study has been an alternative way of collecting behavioral data in natural setting. In the following sections, we describe the process of how we prepare for and conduct virtual visits to share our experiences in the application of remote data collection in natural settings by utilizing the advantages of technology described previously.

As part of a longitudinal study exploring maternal biobehavioral influence on infant brain and behavioral devel-opment, we have been conducting remote virtual visits to collect behavioral observation data on mother-infant interaction and infant adaptive behavior through a Health Insurance Portability and Accountability Act (HIPAA) compliant Zoom platform. We selected Zoom as our video conferencing platform because it offers secure recording and data storage features. Zoom provides real-time encryption of meetings and backup recordings while complying with HIPAA regulations. All procedures were optimized for participants to join using their phones to avoid having to exclude anyone due to not having access to a home computer with a camera and microphone. Mothers were instructed to install the Zoom app on their phone to conduct the virtual visit. In the longitudinal study design, we plan to collect behavioral data when the infants are 3, 6, 18, and 24 months old. We have been conducting remote data collection for the 3- and 6-month-old visits and will conduct in-person data collection for the 18- and 24-month-old visits because laboratory cameras that can adjust angles are better to capture the movement of older infants. For this article, we discuss our approaches to conducting virtual visits with mothers and their 3- and 6-month-old infants; however, tasks and instructions for 6 months are the same except for the specific book and toy provided. We selected age-appropriate books and toys for each time point.

### Preparation for the Virtual Visit

**FIGURE 1:**
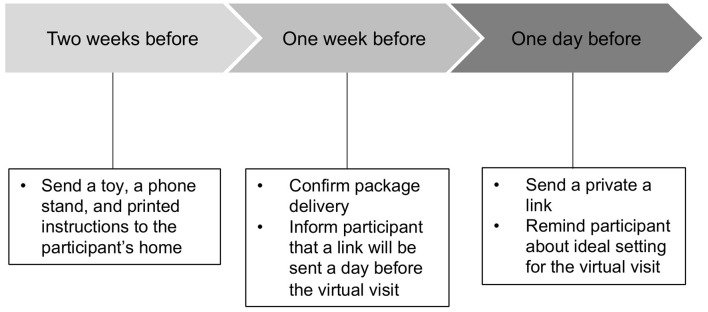
Timeline for virtual visit preparation.

**FIGURE 2:**
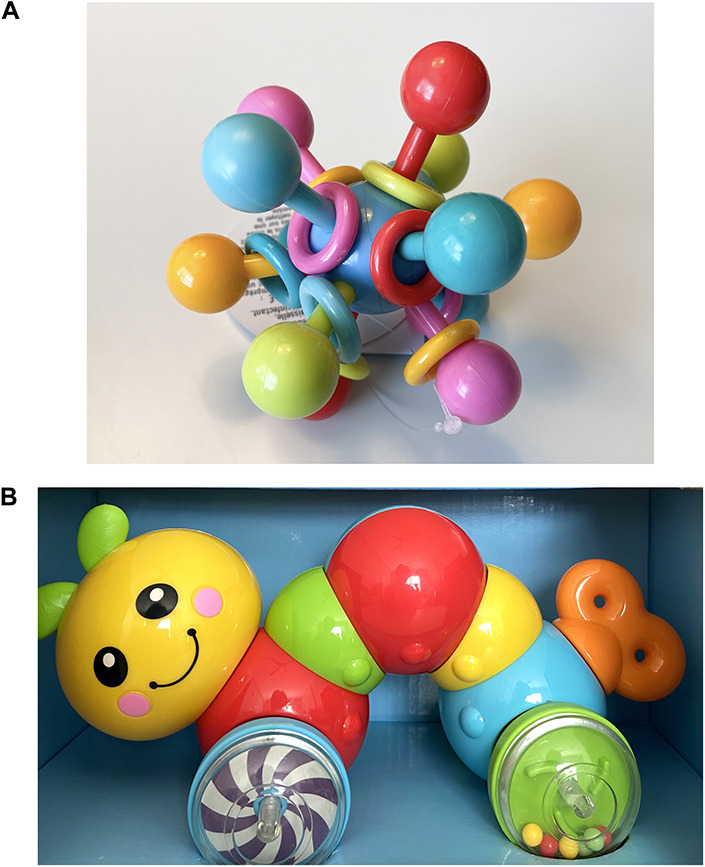
Standardized toys for free play sessions for **(A)** 3-month-old and **(B)** 6-month-old infants.

**FIGURE 3:**
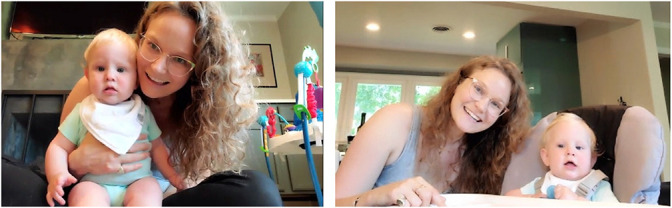
Recommended positions for virtual visit.

Participants were recruited from previous research and participant referral. During the consent call, a trained research assistant explained the general description of tasks and that their interactions will be recorded. Participants were informed that recorded videos will be stored in the institutional data center with restricted permissions and access. Moreover, the research assistant explicitly told the participants that they could withdraw from the study at any time for any reason and it would not be held against them. After the initial consent call, specific preparations for the virtual visit occurred at three points: 2 weeks, 1 week, and 1 day before the virtual visit (Figure 1). Two weeks before the visit, we mailed a toy, a phone stand, and printed instructions to the participant’s home. The toy (Figure 2) was chosen because it is developmentally appropriate and entertaining, would elicit interaction between mothers and infants, families were unlikely to already have it, and it was easy to mail (i.e., it was available on Amazon Prime). In the instructions (see Supplementary Appendix 1), directions for Zoom app installation and example photos of recommended positions the mother and infant should use during the visit were included (Figure 3). Mothers were asked to not show the toy that was sent to their baby until the visit to ensure it remained novel to the infant. One week before the visit, we contacted mothers again to confirm that they received the package, and we informed them that a private Zoom link would be sent a day before the visit. A HIPAA-compliant Zoom link was texted to the mother’s phone a day before the visit. We also reminded her that the session would be recorded and what the ideal setting for the visit would look like (e.g., a quiet and bright place for the visit, ensuring their phone is fully charged, etc.). Because 3-month-old infants have difficulty sitting unsupported, we asked mothers to use a supportive pillow, a Bumbo seat, or a bouncer during the virtual visit.

### During the Virtual Visit

Virtual visits were scheduled with consideration for the infant’s feeding and sleep schedule to ensure that the infant was alert and ready to play during the session. Just before the visit, the experimenters turned off their computer notifications and phones and opened the materials for the visit. Once the session began, but before recording began, the mother was informed that the session was going to be recorded and asked to turn on the “do not disturb” mode on her phone. Recording began once she confirmed that she was comfortable with it. She was then asked to position her phone horizontally using the phone stand provided. After positioning her phone as asked, she was asked to troubleshoot a camera angle and position that would capture both her and her infant’s faces.

**FIGURE 4:**
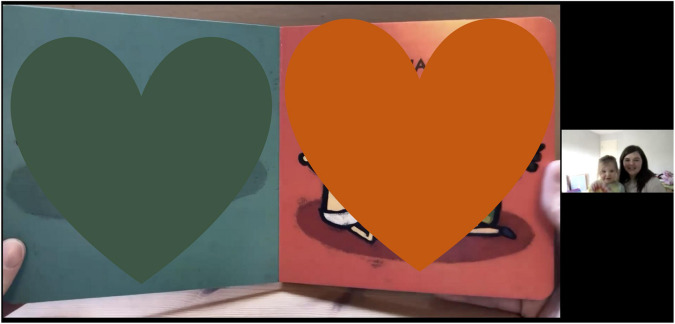
Issue with size of participant’s screen in Zoom.

**FIGURE 5:**
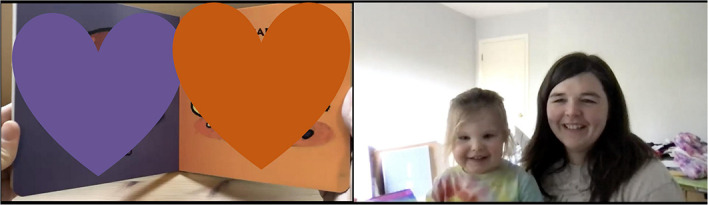
Adjusting size of participant’s screen in Zoom.

**FIGURE 6:**
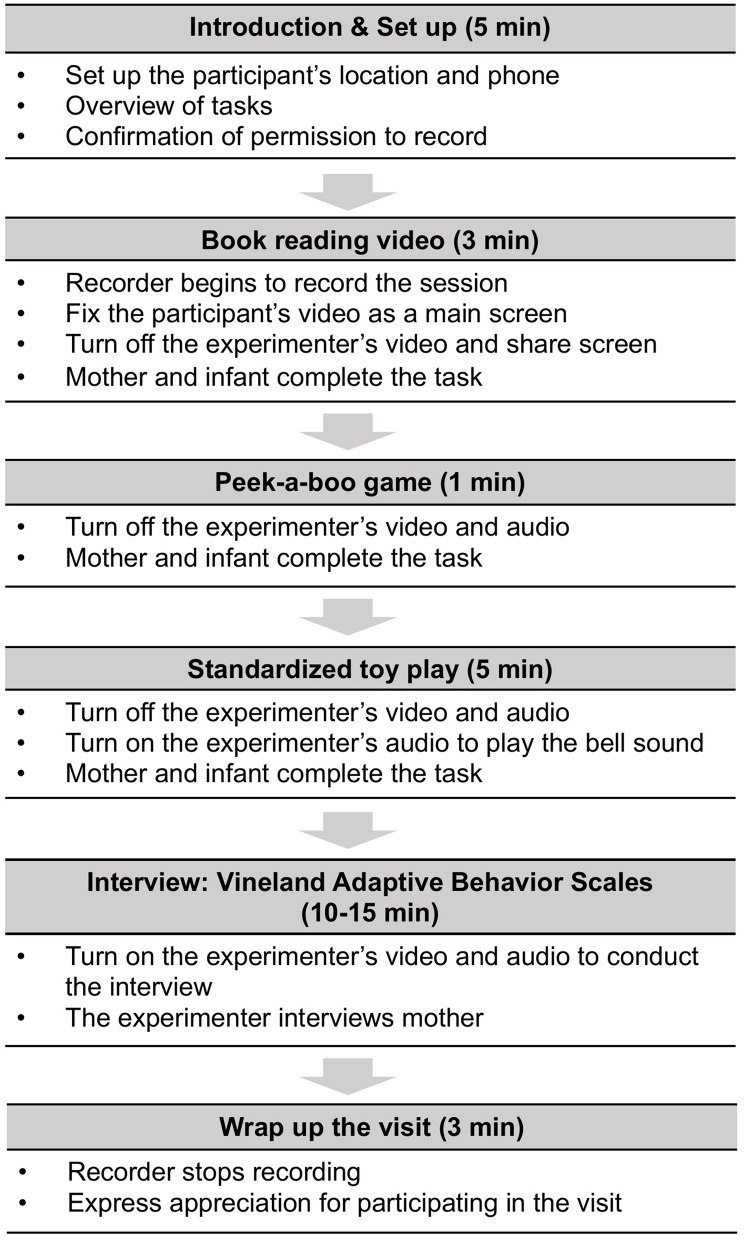
Process of virtual visit.

Two research assistants were required for each visit: one research assistant (the experimenter) ran the tasks while the other research assistant (the recorder) recorded the visit. Two experimenters were necessary because during a Zoom meeting in which a person is sharing their screen (in this case, the experimenter), the person sharing their screen is unable to adjust the size of the window showing the participant’s video (Figure 4). The recorder was responsible for maximizing the size of the window displaying the participant’s video during the session (Figure 5). During the visit, the two research assistants worked together to complete the three tasks including the book reading video, peek-a-boo game, and toy play and removal to assess mother-infant interaction. The experimenter provided a general description of the tasks followed by specific instructions before each task. In addition, the Vineland Adaptive Behavior Scales – Third Edition (VABS-III; Sparrow et al., 2016), a semi structured parent interview was designed to measure the infant’s adaptive behavior (see Figure 6 for the visit schedule). If at any time the infant became fussy or needed a break, the experimenter allowed a break to calm the infant down. The pace of the visit was determined by the mother-infant dyad’s needs and conditions.

#### Book Reading Video

**FIGURE 7:**
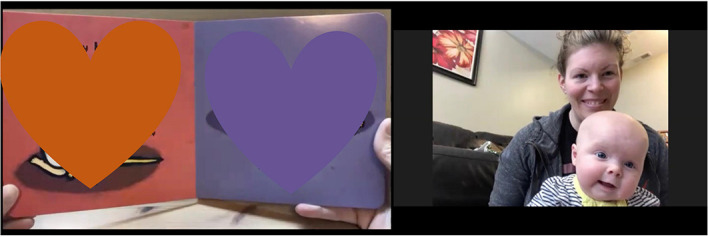
Data from a book reading video.

Mothers and their infants were asked to watch a 2-min video of the book “*Happy Baby, Sad Baby*” by Leslie Patricelli, being read aloud by a female research team member (see Supplementary Appendix 2). In the video participants could not see the face of the reader, but could see her hands turning the pages as she read. We chose to have an experimenter read the book instead of mothers to ensure that all infants received the same stimulation related to the book, allowing for a standardized measure of attention (i.e., time spent attending to the book vs. looking away from the book). The mother was asked to sit with her infant in her lap while they watched the video (Figure 7). Mothers were instructed not to redirect their infant’s attention should they turn away or otherwise stop attending to the video to allow for robust and accurate quantification of infant attention directed toward the video. The experimenter then shared their screen to show the book reading video and turned off their own camera. After the video was over, the mother was prompted to talk to her infant about the book for 1 min. Recordings were labeled using a study ID number free of personally identifiable information. Coding of the interaction will take place at a later time and will include assessments of maternal behavior (i.e., maternal sensitivity and responsiveness) and infant attentiveness (e.g., time spent attending to the video). To account for the confounding effects of screen exposure on infant behavior, we also asked mothers about their infants’ exposure to screen media at the end of the visit.

#### Peek-a-Boo Game

**FIGURE 8:**
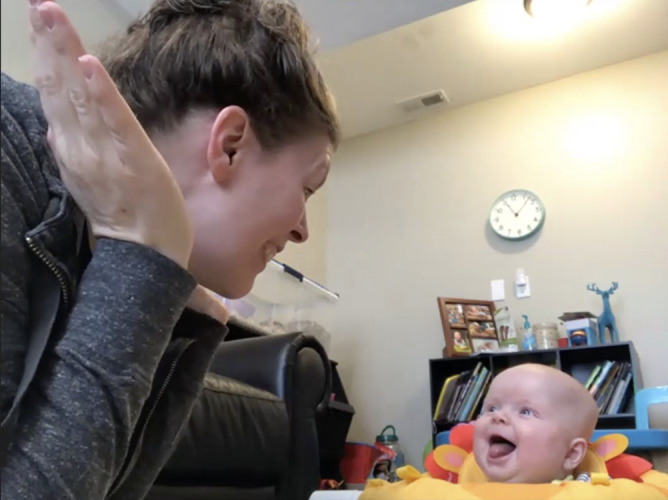
Data from a peek-a-boo game.

The experimenter then asked the mother to initiate and participate in a 1-min play session of peek-a-boo with her infant (Figure 8). At the 3- and 6-month visits, the dyad sat on the floor with the infant supported with pillows or in a highchair if available (Figure 3 for recommended positions). Once the mother began the peek-a-boo game, the experimenter turned off their camera and muted their audio. This interaction will also be coded at a later time for infant positive and negative affect and maternal sensitivity and intrusiveness.

#### Standardized Toy Play

**FIGURE 9:**
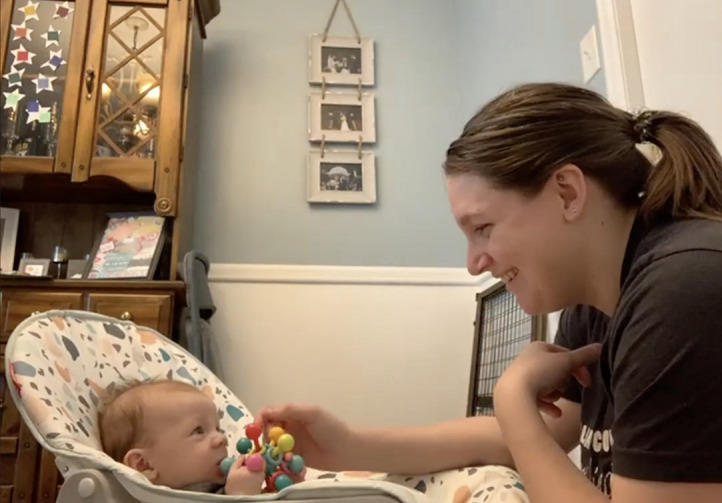
Data from a standardized toy play.

Mothers and their infants participated in a 2-min play session using the toy that was sent earlier, followed by toy removal for 1 min, and another 2-min play session. Each mother was asked to interact with her infant as she normally would (Figure 9). Once the mother began to play with her infant, the experimenter turned off their camera and muted their audio, and a 1-min timer was started. Following the toy play session, the mother was instructed to remove the toy from her infant, but to leave it where her infant could see, but not reach it, for 1 min. After 1 min, the mother was prompted to give the toy back to her infant and to play for two additional minutes. Infant positive and negative affect and maternal sensitivity and intrusiveness will be coded at a later time.

#### Infant’s Adaptive Behavior

The Vineland Adaptive Behavior Scales – Third Edition (VABS-III; Sparrow et al., 2016) assessed child adaptive behavior in several developmental domains: communication, socialization, daily living skills, and motor. The VABS-III was designed to be administered by an experimenter as a semi-structured interview with a caregiver, in this case the mother. The interview usually takes 10–15 min to conduct. Because the infant portions of the session were completed, infants could stay or go to another caregiver during the interview. The VABS-III consists of behaviors that infants display without physical help or reminders. Both the experimenter and the recorder were trained to administer this measure, and both rated the mother’s answers using a printed questionnaire. We used Pearson’s web-based system (Q-global) for test administration and scoring. Q-global supports both management of examinees’ records and production of specific and comprehensive reports of automatically calculated scores. After the experimenter entered their scores for each item in Q-global, the recorder verified the scores that the experimenter entered and published a total score report.

## Challenges and Recommendations

In this section, we describe difficulties that we encountered, and recommendations and considerations that facilitated remote data collection in the home setting using video conferencing tools. First, researchers need to confirm that the participant’s technological environment is sufficient for remote data collection. Because synchronous video conferencing tools use the internet; therefore, stable internet connectivity and quality are the first requirements for both the research team and participants to collect data remotely using the procedures described in this manuscript. Most video conferencing platforms used on phones work an average internet speed between 60 and 100 kbps (McNally, 2020). In addition, other devices and phone capabilities including microphones and cameras should be checked before the visit. Researchers can check the participant’s technological environment during the consent session, or can schedule a separate practice session in the same setting as the visit. If the participant’s technological environment is insufficient for remote data collection, the research team can offer technological aids. For example, if the quality of the internet connection is insufficient, the research team can lend the participant a Wi-Fi hotspot or other devices to meet technological requirements. In addition, there are many public libraries that loan Wi-Fi hotspots to community members.

Second, an instruction document or checklist of logistical set up requirements helps participants prepare for the virtual visit. Unlike the laboratory setting, researchers are less able to control the space where participants’ behavior is observed. We ask participants to find a quiet and uninterrupted place for the visit, charge their phone, and turn on “do not disturb” mode on their phones. A simple instruction booklet including pictures facilitated participants’ set up of logistical requirements (see Instructions in Supplementary Material). In particular, example pictures of good camera angles and providing a phone stand with markings of specific angles aided participants in following recommended settings (see Figure 3). Moreover, a short tutorial video may facilitate standardized participation. For example, the Emerging Minds Lab at Arizona State University shared a tutorial video for a cognitive task via Twitter^5^.

Third, as mentioned earlier, an effective way to ensure the virtual setting will work for the visit is by running practice sessions. Because device malfunction, video or audio issues, and lack of Zoom experience can disrupt remote data collection, practice sessions prepare a research team to set up web-based remote data collection. We have had several mock virtual visits among research team members and two pilot sessions with mother-infant dyads before we started to collect remote data. It was through a pilot session that we discovered the screen proportion issue described previously when the experimenter shared their screen. In the book reading task, we needed to ensure the participant’s screen was large enough to code their behavior. However, once the experimenter shared their screen while recording the session, the shared screen was bigger than the participant’s screen, even though we pinned the participant’s screen (Figure 4). We consulted with the “Many Babies-At Home (MBAH)” group, and a researcher from MBAH proposed an applicable solution to address this issue. To adjust the participant’s screen size, another research assistant (the recorder) joined the session to record the visit. Since the recorder did not share their screen, the recorder was able to make the participants’ face as large as possible (Figure 5). Because remote data collection is currently underutilized as an observational data tool, sharing challenges with other researchers is a productive way to resolve issues and advance methodological skills.

Fourth, researchers should assure that video conferencing platforms offer privacy and secure service for confidentiality of data. Due to significant increases in video conference meetings during the COVID-19 pandemic, uninvited outsiders have more opportunities to enter meetings and interrupt the session (e.g., Zoom bombing). To protect the session and participants, meeting access should be protected by a password, or a research team can use features which control the attendee’s entrance and terminate meeting sessions (i.e., the waiting room feature). Moreover, HIPAA established privacy and security standards must be maintained to protect personal privacy. Researchers need to ensure that the video conferencing platforms utilized provide HIPAA-compliant services. For example, the HIPAA-compliant version of Zoom uses safeguards to prevent any unauthorized access in their environment to meet these HIPAA requirements. Other than privacy and secure service for confidentiality of data, the principles of ethical issues in online data collection are similar to in-person contexts. Lobe et al. (2020) mentioned that ‘‘researchers who already have approval their review board will probably only need to file a simple amendment to their original proposal to shift from in-person to online data collection’’ (p. 5). Common ways to obtain participant’s consent for remote data collection are consent phone call or conference call and email consent form to participants. Research teams ask for scanned signatures or use electronic signature programs such as DocuSign^6^ to collect participants’ signatures. For example, we explained study protocols and answer questions that participants had during a consent call and send a link to a consent form which participant can then sign. During a consent call, our participants were informed about the recording of the sessions, the private and secure data storage, and their right to withdraw from the study at any time for any reason without penalty.

## Discussion

Prior to social distancing guidelines, which led to challenges for inviting participants into the lab, we planned to observe mother-infant interactions and assess infants’ development in person. However, in the COVID-19 era, we needed to find alternative ways to pursue answers to important and pressing research questions. Video conferencing platforms are able to concurrently record back-and-forth exchanges of interactions in a private internet setting. Additionally, time and location flexibility allowed us to consider mothers and infants’ schedules at home. We were able to adjust our observational measures to include synchronous behavioral assessments and a semi-structured interview with mothers to collect infants’ developmental information without any attrition so far. Visual inspection suggests that the quality of data obtained through the virtual visit has been similar to data obtained from the lab setting; mother and infants faces are clearly visible, allowing for consistent coding, and the same study materials were used across participants (see Figures 7–9). When participants veered away from prescribed camera angles or protocols, an experimenter guided mothers to conduct the study in a consistent way across all participants. A link to an example video of a 3-month virtual visit is provided in Supplementary Appendix 3. As we experienced, shifting from traditional face-to-face data collection to remote data collection required careful consideration of conceptual and logistical aspects of data collection. In this report, we describe the application of remote data collection in a natural home setting through a video conferencing platform to share our experiences with researchers who are considering new remote data collection methods.

Because remote data collection through video conferencing platforms is still a nascent topic, there are limitations and careful considerations for future research. For example, web-based platforms require digital tools and knowledge and internet connectivity. These requirements might overlook populations that lack access to technology tools or confidence in using them. Even though the digital environment has been rapidly developing, it is important to consider underrepresented groups who struggle with technology to gain generalizable knowledge. In a qualitative study using Zoom (Archibald et al., 2019), most participants encountered some challenges with joining the session. Researchers need to support the use of technology with approachable instructions and tools, as demonstrated here. Another limitation might be that families may not want to participate from their homes for a variety of reasons. Because home environments reflect families’ lifestyles, there may be participants who do not want to share this view into their homes. In this case, researchers can suggest other places, such as public libraries, for families to participate. Although it may vary depending on the library, most libraries offer private rooms for community members to reserve.

Here we only focused on using the HIPAA-compliant version of the Zoom platform. Practical features and considerations could be different depending on videoconferencing platforms. It is important to consider the functions that will best convey a project’s needs and institutional support. There are several options for remote data collection. For example, Webex^7^ has also been widely used for research and Skype^8^ is common for interpersonal communication. Researchers could also utilize the HD video feature through GoToMeeting^9^. Lobe et al. (2020) proposed the following criteria be considered when choosing a videoconferencing platform: “the number of participants in a same session, audio/video recording, one-click access for participants, and privacy features (p. 3).” Researchers also need to find secure data storage in accordance with ethical procedures. Prior literature has recommended that recording data through the internet should be saved in local storage (i.e., the researcher’s computer) and not the cloud storage provided by the platform to preserve the third party’s privacy (Lobe et al., 2020).

Despite limitations, remote data collection through videoconferencing platforms offers opportunities for researchers to pursue data collection until social distancing recommendations are relaxed, and beyond. Significant increases in access to electronic devices and the internet across the world, improvements in the platforms, and sharing practical guidelines among researchers promise to advance the effective use of remote data collection. Researchers can increase rigor by utilizing advantages of technologies, detailed and approachable instructions with careful considerations, practice sessions, and electronic safeguards. It is our hope that sharing our experiences and issues in remote data collection with mothers and infants with other researchers will extend methodological tools to historically underrepresented populations (i.e., rural populations) in developmental science.

## Data Availability Statement

The original contributions presented in the study are included in the article/Supplementary Material, further inquiries can be directed to the corresponding author.

## Ethics Statement

The studies involving human participants were reviewed and approved by the Virginia Polytechnic Institute and State University. Written informed consent to participate in this study was provided by the participants’ legal guardian/next of kin. Written informed consent was obtained from the individual(s) for the publication of any identifiable images or data presented in this article.

## Author Contributions

ES: protocol design, data collection, study execution, and writing – original draft preparation. CS: protocol design and writing – reviewing and editing. BH: protocol design, writing – reviewing and editing, project administration, and funding acquisition. All authors contributed to the article and approved the submitted version.

## Conflict of Interest

The authors declare that the research was conducted in the absence of any commercial or financial relationships that could be construed as a potential conflict of interest.

## Publisher’s Note

All claims expressed in this article are solely those of the authors and do not necessarily represent those of their affiliated organizations, or those of the publisher, the editors and the reviewers. Any product that may be evaluated in this article, or claim that may be made by its manufacturer, is not guaranteed or endorsed by the publisher.

## Footnotes

1
github.com/orgs/lookit/projects/

2
discoveriesonline.org

3
https://manybabies.github.io/MB-AtHome

4
https://zoom.com

5
https://twitter.com/EmergingMindsAZ/status/1281618737079017473?s=20

6
https://www.docusign.com

7
www.webex.com

8
www.skype.com

9
www.gotomeeting.com
