# Supplementary material for: Advances in Behavioral Remote Data Collection in the Home Setting: Assessing the Mother-Infant Relationship and Infant’s Adaptive Behavior via Virtual Visits
Source: Front Psychol. 2021 Oct 1;12:703822. doi: 10.3389/fpsyg.2021.703822 (PMC8517484; doi:10.3389/fpsyg.2021.703822)
Supplement: Supplementary file 1 [file Data_Sheet_1.docx]

**Appendix 1**

**Appendix 2**

A link to a video of book reading:

<https://drive.google.com/file/d/1Att2QPPtpwNDaDwhB-IxzIjBkWGYYTzd/view?usp=sharing>

**Appendix 3**

A link to an example video of virtual visit:

https://drive.google.com/file/d/1ww3Rxd4vj4K1GCaYT-l7r1fhByz6_k3B/view?usp=sharing
